# Prediction of the Responsiveness to Vagus-Nerve Stimulation in Patients with Drug-Resistant Epilepsy via Directed-Transfer-Function Analysis of Their Perioperative Scalp EEGs

**DOI:** 10.3390/jcm11133695

**Published:** 2022-06-27

**Authors:** Dongyeop Kim, Taekyung Kim, Yoonha Hwang, Chae Young Lee, Eun Yeon Joo, Dae-Won Seo, Seung Bong Hong, Young-Min Shon

**Affiliations:** 1 Department of Neurology, Seoul Hospital, Ewha Womans University College of Medicine, Seoul 07804, Korea; hap2028@ewha.ac.kr; 2 Department of Medical Device Management and Research, Samsung Advanced Institute for Health Sciences and Technology (SAHIST), Sungkyunkwan University, Seoul 06355, Korea; t0.kim.smc@gmail.com; 3 Biomedical Engineering Research Center, Samsung Medical Center, Seoul 06351, Korea; 4 Department of Neurology, The Catholic University of Korea Eunpyeong St. Mary’s Hospital, Seoul 03312, Korea; yoonhaha10@gmail.com; 5 Department of Neurology, Samsung Medical Center, Sungkyunkwan University School of Medicine, Seoul 06351, Korea; feelsogood1002@gmail.com (C.Y.L.); ejoo@skku.edu (E.Y.J.); daewon@skku.edu (D.-W.S.); sbhong@skku.edu (S.B.H.)

**Keywords:** drug-resistant epilepsy, vagus-nerve stimulation, electroencephalography, brain connectivity, directed transfer function

## Abstract

This study aims to compare directed transfer function (DTF), which is an effective connectivity analysis, derived from scalp EEGs between responder and nonresponder groups implanted with vagus-nerve stimulation (VNS). Twelve patients with drug-resistant epilepsy (six responders and six nonresponders) and ten controls were recruited. A good response to VNS was defined as a reduction of ≥50% in seizure frequency compared with the presurgical baseline. DTF was calculated in five frequency bands (delta, theta, alpha, beta, and broadband) and seven grouped electrode regions (left and right frontal, temporal, parieto-occipital, and midline) in three different states (presurgical, stimulation-on, and stimulation-off states). Responders showed presurgical nodal strength close to the control group in both inflow and outflow, whereas nonresponders exhibited increased inward and outward connectivity measures. Nonresponders also had increased inward and outward connectivity measures in the various brain regions and various frequency bands assessed compared with the control group when the stimulation was on or off. Our study demonstrated that the presurgical DTF profiles of responders were different from those of nonresponders. Moreover, a presurgical normal DTF profile may predict good responsiveness to VNS.

## 1. Introduction

Epilepsy is a common neurological disease with an overall incidence of approximately 61.4 per 100,000 person-years [1]. Among patients with epilepsy (PWEs) who are receiving antiseizure medication, approximately one-third remain refractory to drug therapy [2,3]. When diagnosed with drug-resistant epilepsy, surgical intervention should be considered a treatment option [4]. Vagus-nerve stimulation (VNS) has been approved for the treatment of epilepsy without age or seizure-type restrictions in most countries and is widely performed when resective surgery is not applicable or not completely effective [5]. Although VNS was introduced in the clinical field decades ago, its mechanism of action and the prediction of its efficacy preoperatively remain under investigation.

The reported efficacy of VNS varies. The percentage of responders whose seizure frequency has been reduced by ≥50% from the baseline postoperatively has been reported to range from 23% to 45% in randomized controlled trials [6,7,8]. In the first meta-analysis of the efficacy of VNS on epilepsy, Englot et al. reported that seizure frequency was reduced by an average of 45% and seizures were reduced by ≥50% in approximately 50% of the patients after VNS [9]. Considering that approximately 50% of the patients who receive VNS implantation may not experience any effect from the surgery, the lack of predictive factors leads to hesitation regarding the performance of this surgery. Thus, the current VNS treatment is far from personalized medicine.

Biomarkers including connectomic indicators, electrophysiological features, neuroimaging findings, and systemic biomarkers have been reviewed as factors that can be used to predict the effects of VNS [10]. As PWEs undergo various clinical tests, including EEG, magnetoencephalography (MEG), functional MRI (fMRI), diffusion tensor imaging, and 18F-fluorodeoxyglucose positron emission tomography (18F-FDG PET), depending on their clinical settings, these modalities generate data for connectomics studies. Because epilepsy is considered a network disorder [11] and the vagus nerve projects to various brain regions through the nucleus tractus solitarius, biomarkers related to network-based connectivity are thought to be promising in this setting. Studies using fMRI identified areas, including the thalamus, that have greater connectivity in the responder group, whereas one study using 18F-FDG PET detected several brain regions (brainstem, cingulate gyrus, cerebellum, bilateral insula, and putamen) with greater nodal strength in the nonresponder group [12,13]. Although fMRI and 18F-FDG PET provide good spatial resolution, the directionality of connectivity cannot be obtained using these imaging modalities. Functional integration can be studied on the basis of functional and effective connectivity [14]. Functional connectivity is defined as the statistical dependencies among remote neurophysiological events, whereas effective connectivity refers explicitly to the effect that one neural system exerts over another, either at a synaptic or population level [15]. Biomarkers using functional connectivity analysis with electrophysiological activity have been applied to determine responsiveness to VNS treatment [10], and effective connectivity analysis was introduced on scalp EEG in a previous study using partial directed coherence [16]. Directed transfer function (DTF), as a type of function for effective connectivity analysis, is a multichannel parametric method based on an autoregressive model that is used to determine the patterns of neural information flow in PWEs [17].

Herein, we present a study that was performed using presurgical and postsurgical scalp EEG in PWEs who underwent VNS and had a clear-cut clinical response. This study aims to compare effective connectivity measures of scalp EEG between responder and nonresponder groups implanted with VNS.

## 2. Materials and Methods

### 2.1. Participants

Twelve patients (age: 33.7 ± 10.2 years, ten men and two women) with drug-resistant epilepsy were selected among the 32 individuals who had VNS implantation surgery (Cyberonics, Houston, TX, USA) between 2014 and 2015 at the Samsung Medical Center, Korea. Additionally, ten controls (age: 35.1 ± 17.3 years, five men and five women) who had not been diagnosed with nervous-system or psychiatric diseases were recruited as the control group. The patients that were included in this study were (1) those who could clearly report their seizure frequency; (2) those for whom EEG data were available with appropriate quality for connectivity analysis; and (3) those with >5 years of postoperative follow-up.

All patients underwent comprehensive presurgical evaluation including video EEG monitoring, 3T brain MRI, 18F-fluorodeoxyglucose PET, ictal and interictal cerebral blood-flow-based single-photon emission CT, Wada test, and neuropsychological test. On the basis of the test results, a patient-management conference was held to discuss the therapeutic strategy for each patient, and VNS was chosen as an alternative option to resective surgery. Patients had been taking 4 to 6 antiseizure medications before the surgery, and there were no, or minor, medication changes after surgery. This study was approved by the Institutional Review Board of the Samsung Medical Center (IRB No. 2016-04-103).

### 2.2. VNS Parameters and Responsiveness

We defined responsiveness to VNS as a reduction in seizure frequency, at the time of the 5-year follow-up, of ≥50% compared to the baseline. Six patients (age: 37.5 ± 10.99 years, five men and one woman) were classified as responders, whereas the remaining six patients (age: 29.8 ± 8.59 years, five men and one woman) were classified as nonresponders. After the VNS implantation, a stimulator generated stimuli in a set of 30-s on and 5-min off conditions. For all patients, the EEG recordings were performed at three different time points, i.e., presurgical state, during the stimulation-on state, and during the stimulation-off state.

### 2.3. EEG Recording

Scalp EEG was recorded using a 19-channel NicoletOne EEG system (Natus Medical Inc., Pleasanton, CA, USA) according to the international 10–20 system before and after VNS surgery. Presurgical routine EEG was performed 3 months before the VNS implantation, and follow-up EEG was performed for analysis after the surgery. EEG signals were collected during the artifact-free resting state with eyes closed; these signals were obtained at least 24 h apart from the seizure, and drowsy or sleeping states were excluded. The signal was sampled at a rate of 500 Hz and band-pass filtered between 0.5 and 70 Hz. For analysis, 20 segments of 2-s, noise-free EEG epochs were extracted from each dataset by two epileptologists (Lee C.Y. and Shon Y.M.).

### 2.4. Connectivity Analysis

The raw EEG signal was loaded into MATLAB R2018a, and the resulting data were processed using scripts written in-house on the basis of the study of Omidvarnia et al. [18]. Subsequently, for effective connectivity analysis, we applied a short-time-based DTF. The model order for the DTF measure using time-varying data was estimated optimally using the ARfit package [19] and Schwarz’s Bayesian criterion [20]. DTF was calculated for all combinations of the 19 channels for every 0.5-Hz interval from 1 to 50 Hz. The frequency bands were divided as follows: delta (0.5–4 Hz), theta (4–8 Hz), alpha (8–12 Hz), beta (12–30 Hz), and broadband (0.5–30 Hz). The gamma band (30–50 Hz) power was excluded from the analysis because of concerns about contamination with artifacts [21,22]. The connectivity measures from 19 channels were grouped into seven nodes, as follows: left frontal (Fp1, F3, and C3), left temporal (F7, T7, and P7), left parieto-occipital (P3 and O1), right frontal (Fp2, F4, and C4), right temporal (F8, T8, and P8), right parieto-occipital (P4 and O2), and midline (Fz, Cz, and Pz). Permutation tests were applied to the DTF values to check statistical significance (which was set at *p* < 0.05). Any values that did not meet the significance criterion were removed from the remainder of the analysis. After permutation tests, the median of the resulting matrices of DTF values were calculated for all nodes across frequencies and the flow of information between each pair of nodes in each frequency band was indicated. The amount of information flowing toward and from the nodes was termed inflow and outflow, respectively.

The connectivity values of the brain network are represented in a graph consisting of nodes and edges connecting them. The strength of the information flow is indicated by the color of the edges. Each circle denotes a node, i.e., a group of adjacent electrodes, and the diameter of each circle represents nodal strength, which is the sum of all inflow and outflow connectivity measures of the node. The color of the node represents the direction: blue for inflow and red for outflow connectivity. A significant change in connectivity compared with the control group is indicated by a thickened circle.

### 2.5. Statistical Analysis

First, the connectivity measures of each patient in each state, i.e., the presurgical, stimulation-on, and stimulation-off states, were compared with respect to the control. Second, we assessed the significance of differences in connectivity between the two groups for each connection and node, for each frequency band. Third, to determine the effect of VNS, we tested the changes in connectivity in the stimulation-on and stimulation-off states compared with those in the presurgical state. All comparisons were calculated using the Wilcoxon rank-sum test. The statistical results were corrected using Bonferroni correction for multiple comparisons. Statistical analyses were performed using MATLAB R2018a. All calculated *p* values were two tailed, and statistical significance was set at *p* < 0.05.

## 3. Results

### 3.1. Patient Characteristics

Twelve patients were retrospectively included in this study according to the aforementioned criteria (Table 1). The mean age at surgery was 33.7 ± 10.2 years with mean epilepsy duration of 21.1 ± 8.4 years.

### 3.2. Presurgical Effective Connectivity Analysis Using DTF

**Figure 1:**
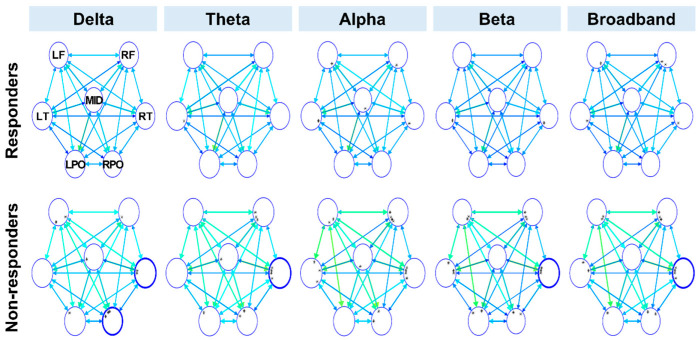
Presurgical inflow connectivity analysis of the responder and nonresponder groups compared with the control. Note that each circle denotes a node and that the diameter of the circle represents nodal strength, which is the sum of all connections toward the node. The strength of the information flow is indicated by the color of the edges. Thickened circles represent nodes with significantly greater inflow connectivity compared with those of the control. LF—left frontal; RF—right frontal; LT—left temporal; RT—right temporal; LPO—left parieto-occipital; RPO—right parieto-occipital.

**Figure 2:**
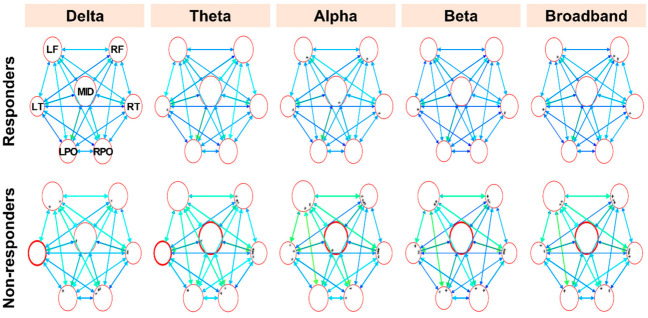
Presurgical outflow connectivity analysis of the responder and nonresponder groups compared with the control. The thickened circles represent nodes with a significantly greater connectivity from the node compared with those of the control. LF—left frontal; RF—right frontal; LT—left temporal; RT—right temporal; LPO—left parieto-occipital; RPO—right parieto-occipital.

**Figure 3:**
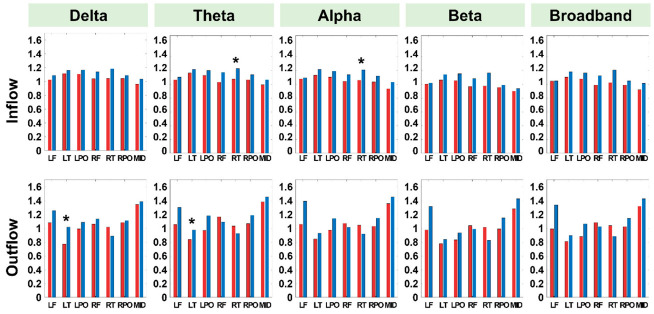
Comparison of the inflow and outflow connectivity measures between the responder and nonresponder groups in the presurgical state. The asterisk denotes a statistically significant difference between the two groups.

The presurgical connectivity analysis demonstrated that the responder group had a normal connectivity profile in terms of the nodal strength of both the inward and outward flow (Figure 1 and Figure 2). Conversely, the nonresponder group showed increased inward strength in the right temporal region (delta, theta, beta, and broadband frequencies) and right parieto-occipital region (delta frequency); and increased outward strength in the left temporal region (delta and theta frequency) and midline region (theta, alpha, beta, and broadband frequency), compared with the control group (Figure 1 and Figure 2). Among these regions, the nonresponder group showed a statistically significant increase in inflow connectivity in the right temporal region (theta and alpha frequencies) and increased outflow connectivity in the left temporal region (delta and theta frequencies) compared with the responder group (Figure 3).

### 3.3. Effect of VNS Implantation on Effective Connectivity

There was no noticeable connectivity change after VNS implantation compared with that in the presurgical state in each group and in each state, with the exception of the increased inward connectivity in the alpha frequency band detected in the left frontal region of the responders when the stimulation was turned on. No significant connectivity change was noted in the outward connectivity analysis or in the stimulation-off state compared with the presurgical state. More detailed results are provided in Supplementary Figures S1 and S2.

Similarly to the results of the presurgical DTF analysis, the nonresponder group had increased inward and outward connectivity measures in the various brain regions and in the various frequency bands examined compared with the control group when the stimulation was on or off (Supplementary Figures S1 and S2). In the responder group, only the inflow to the right parieto-occipital region (alpha frequency, stimulation-on state) and the outflow from the left parieto-occipital region (alpha frequency, stimulation-on and stimulation-off states) were significantly higher than those in the control group, whereas other brain regions showed normal connectivity (Supplementary Figures S1 and S2). A direct comparison between the responder and nonresponder groups regarding the stimulation-on or -off states revealed significantly higher connectivity in the nonresponder group than in the responder group (Supplementary Figures S3 and S4).

## 4. Discussion

We aimed to evaluate effective connectivity measures to predict responsiveness to VNS in patients with refractory epilepsy. To the best of our knowledge, this is the first study to elucidate the effectiveness of DTF for demonstrating not only connectivity changes, but also whether it can be applied as an early prognostic marker after VNS therapy. In this study, we applied DTF analysis in three different states, i.e., the presurgical, stimulation-on, and stimulation-off states. Indirect comparison analyses were conducted in the responder and nonresponder groups separately vs. the connectivity observed in the control group; subsequently, a direct comparison of connectivity measures between the responder and nonresponder groups was performed. Additionally, we focused on the following investigations: (1) presurgical indirect and direct comparisons; (2) the detection of connectivity changes from the presurgical to the stimulation-on or stimulation-off states within the group, and (3) comparison of the connectivity after VNS between the responder and nonresponder groups. The results corresponding to each question posed in this study were as follows: (1) the responder group had a DTF connectivity profile close to that of the control group, whereas the nonresponder group showed increased inward and outward nodal strength in various regions and frequency bands; (2) no consistent DTF connectivity change was observed after VNS implantation compared with that in the presurgical state; and (3) the increased inflow and outflow DTF connectivity profiles detected in the various regions and frequency bands persisted in the nonresponder group after VNS implantation, whereas the responder group showed increased inward strength only in the right parieto-occipital region, and augmented outward strength only in the left parieto-occipital region in the stimulation-on state.

For the prediction of responsiveness to VNS, it is essential to identify an appropriate prognostic metric that can be recorded preoperatively. Although Fraschini et al. reported a lack of differences in the phase lag index (PLI) between the responder and nonresponder groups before VNS [21], other studies did not report the presurgical PLI values [23,24]. Babajani-Feremi et al. first investigated the prediction of VNS outcome based on MEG data acquired before the implantation of VNS, and revealed that the nonresponders exhibited a higher transitivity and lower modularity derived from graph measures than the responders [25]. Moreover, DTF has been mainly used to demonstrate the propagation of seizures or the seizure onset zone [17,26,27]. One study that performed a network analysis using a directed connectivity measure, i.e., partial directed coherence, showed decreased network efficiency during sleep after acute VNS in responders, but not in nonresponders [16]. Enhanced synchronization in epileptic networks has been studied well in previous reports using EEG and MEG [28,29,30,31]. Therefore, a presurgical directed connectivity profile close to the control group may predict a good response regarding seizure reduction after VNS implantation.

Cortical synchronization or desynchronization activity by vagal stimulation was observed in previous animal studies [32,33]. EEG desynchronization has been postulated as a major mechanism of action of VNS [34]. By applying a functional connectivity analysis based on the results of a neurophysiological test, global desynchronization has been proven as a potential mechanism of action of VNS in humans. Fraschini et al. first reported that VNS induced desynchronization in gamma bands, which was correlated with responsiveness to long-term stimulation up to several years using PLI [21]. In contrast, Bodin et al. excluded gamma band analysis based on the concern of contamination by artifacts, and reported that responders had a lower level of synchronization, particularly in the delta and alpha bands at several months after VNS implantation [23]. Using stereotactic EEG, Bartolomei et al. found that only decreased functional connectivity corresponded to the responders [35]. In the present study, we attempted to identify changes in effective connectivity after VNS implantation and compared DTF measures among presurgical, stimulation-on, and stimulation-off states within each responder and nonresponder group. However, no consistent changes in connectivity were detected in the stimulation-on state vs. the stimulation-off state. This result differed from that of the previous literature, which reported global desynchronization with PLI values when the stimulation was on in the responder group compared with those when the stimulation was off [16,23,24]. This finding may be attributed to a different time interval between surgery and the EEG study, or a different methodology of EEG analysis employed in the previous studies compared with ours.

The mechanism of effectiveness of VNS is still not fully understood. Vagus nerve fibers are comprised of approximately 80% afferent sensory fibers that primarily project to the nucleus tractus solitarius, which, in turn, sends fibers to other brainstem nuclei that modulate the activity of the subcortical and cortical circuitry [36,37]. Although it is known that both right and left vagus nerves send an afferent signal to the bilateral thalamic nuclei and cerebral cortices, thus affecting the bilateral functional connectivity [38], previous evidence suggests a cortical afferent signal connection of unilaterality in VNS responders. Ibrahim et al. demonstrated that the enhanced connectivity of the thalami to the anterior cingulate cortex and left insula detected in presurgical resting-state fMRI was associated with good responsiveness to VNS [12]. The same group reported that VNS responders possess a more robust left-lateralized white-matter microstructure, as assessed on the basis of the study of diffusion-tensor imaging, which showed increased fractional anisotropy in the left thalamocortical, limbic, and association fibers than nonresponders, as well as greater connectivity in a functional network encompassing the left thalamic, insular, and temporal nodes [39]. Conversely, the study of Zhu et al. indicated that patients with decreased functional connections in resting-state fMRI between the left hippocampus and bilateral thalamus may show a good response to VNS [40]. Although there is still a lack of research and inconsistency regarding the methods of investigation among the previous studies, including the enrolled participants, algorithms, and datasets, the aforementioned reports suggest that the unilaterality of the stimulation and connectivity profile may be related to the responsiveness to VNS. Our study showed that the responder group had significantly decreased inward connectivity toward the right temporal region and decreased outflow connectivity from the left temporal region compared with the nonresponder group. These results suggest that the temporal lobes are important hubs for functional connectivity. The strength of our study lies in the fact that we demonstrated the potential of directed connectivity analysis for probing the effectiveness of VNS, although a larger sample size and a multichannel EEG analysis are warranted.

This study had several limitations. First, a small number of samples were included in this study. The individuals within one group can have diverse connectivity changes between different states, and the significance of the result can be masked by the small sample size. Second, the epilepsy phenotype of the recruited patients was heterogeneous, thus including both focal and generalized epilepsy, as well as unilateral and bilateral epilepsy. The reasons for undergoing VNS implantation were also heterogeneous; some of the patients had bilateral epilepsy, whereas others were not suitable for resective surgery because of concerns about the involvement of the eloquent area. In particular, directed connectivity analysis may be more susceptible to errors than the undirected connectivity measure, because the lateralization of the epilepsy syndrome would affect the directionality of the effective connectivity measure.

## 5. Conclusions

This study was performed to identify a biomarker for the prediction of good responsiveness to VNS before surgery. We suggest that the presurgical effective connectivity profile of responders may be different from that of nonresponders. Put another way, a presurgical connectivity profile close to normal may predict a good prognosis after VNS implantation. In addition, directed connectivity analysis might be an effective tool to examine the mechanism of action of VNS regarding the unilaterality or directionality of the stimulation. Further research with a larger sample size is needed to identify whether the results show consistent findings.

## Figures and Tables

**Table 1:** Baseline characteristics of the patients enrolled in the study.

Patient No.	Age (Years)	Sex	Disease Duration (Years)	Epilepsy Diagnosis	Pre-VNS Seizure Frequency (per Month)	Mean Seizure Reduction Rate for 5 Years (%)	Responsiveness
1	35	M	29	CLE	90	88	Responder
2	38	F	20	Rt. FLE	1	100	Responder
3	37	M	28	Rt. FPLE	11	−24	Nonresponder
4	55	M	29	Lt. TLE	2	61	Responder
5	21	M	12	IGE	4	69	Responder
6	31	M	10	Both TLE	1.5	−33	Nonresponder
7	37	M	7	Both FTLE	2.5	−7	Nonresponder
8	35	F	26	Lt. FPLE	10	−73	Nonresponder
9	23	M	12	IGE	14	33	Nonresponder
10	35	M	15	Both FLE	35	53	Responder
11	41	M	28	Rt. PLE	3	100	Responder
12	25	M	16	IGE	45	−58	Nonresponder

CLE—central lobe epilepsy; FLE—frontal lobe epilepsy; FPLE—fronto-parietal lobe epilepsy; TLE—temporal lobe epilepsy; IGE—idiopathic generalized epilepsy; FTLE—fronto-temporal lobe epilepsy; PLE—parietal lobe epilepsy.

## Data Availability

Not applicable.

## Supplementary Materials

The following supporting information can be downloaded at: https://www.mdpi.com/article/10.3390/jcm11133695/s1, Figure S1: Dot plots of inflow connectivity values in the various brain subregions and different frequency bands examined in the present study; Figure S2: Dot plots of outflow connectivity values in the various brain subregions and different frequency bands; Figure S3: Bar plots depicting the comparison between the responder and nonresponder groups when the stimulation was on; Figure S4: Bar plots depicting the comparison between the responder and nonresponder groups when the stimulation was off.
