# Supplementary material for: Prediction of the Responsiveness to Vagus-Nerve Stimulation in Patients with Drug-Resistant Epilepsy via Directed-Transfer-Function Analysis of Their Perioperative Scalp EEGs
Source: J Clin Med. 2022 Jun 27;11(13):3695. doi: 10.3390/jcm11133695 (PMC9267399; doi:10.3390/jcm11133695)
Supplement: Supplementary file 1 [file jcm-11-03695-s001.zip › 20220514 VNS connectivity_JCM_3. Supplementary Figure legends.pdf]

Supplementary Figure S1. Dot plots of inflow connectivity values in the various brain subregions and different frequency bands examined in the present study. The asterisk indicates a statistically significant difference between the responder and nonresponder groups. †Statistically significant difference compared with the control. ‡Statistically significant connectivity change from the presurgical state.

Supplementary Figure S2. Dot plots of outflow connectivity values in the various brain subregions and different frequency bands. The asterisk indicates a statistically significant difference between the responder and nonresponder groups. †Statistically significant difference compared with the control. ‡Statistically significant connectivity change from the presurgical state.

Supplementary Figure S3. Bar plots depicting the comparison between the responder and nonresponder groups when the stimulation was on. The asterisk denotes a statistically significant difference of values between the two groups.

Supplementary Figure S4. Bar plots depicting the comparison between the responder and nonresponder groups when the stimulation was off. The asterisk denotes a statistically significant difference of values between the two groups.
