# Supplementary figures and images for: Prediction of the Responsiveness to Vagus-Nerve Stimulation in Patients with Drug-Resistant Epilepsy via Directed-Transfer-Function Analysis of Their Perioperative Scalp EEGs
Source: J Clin Med. 2022 Jun 27;11(13):3695. doi: 10.3390/jcm11133695 (PMC9267399; doi:10.3390/jcm11133695)

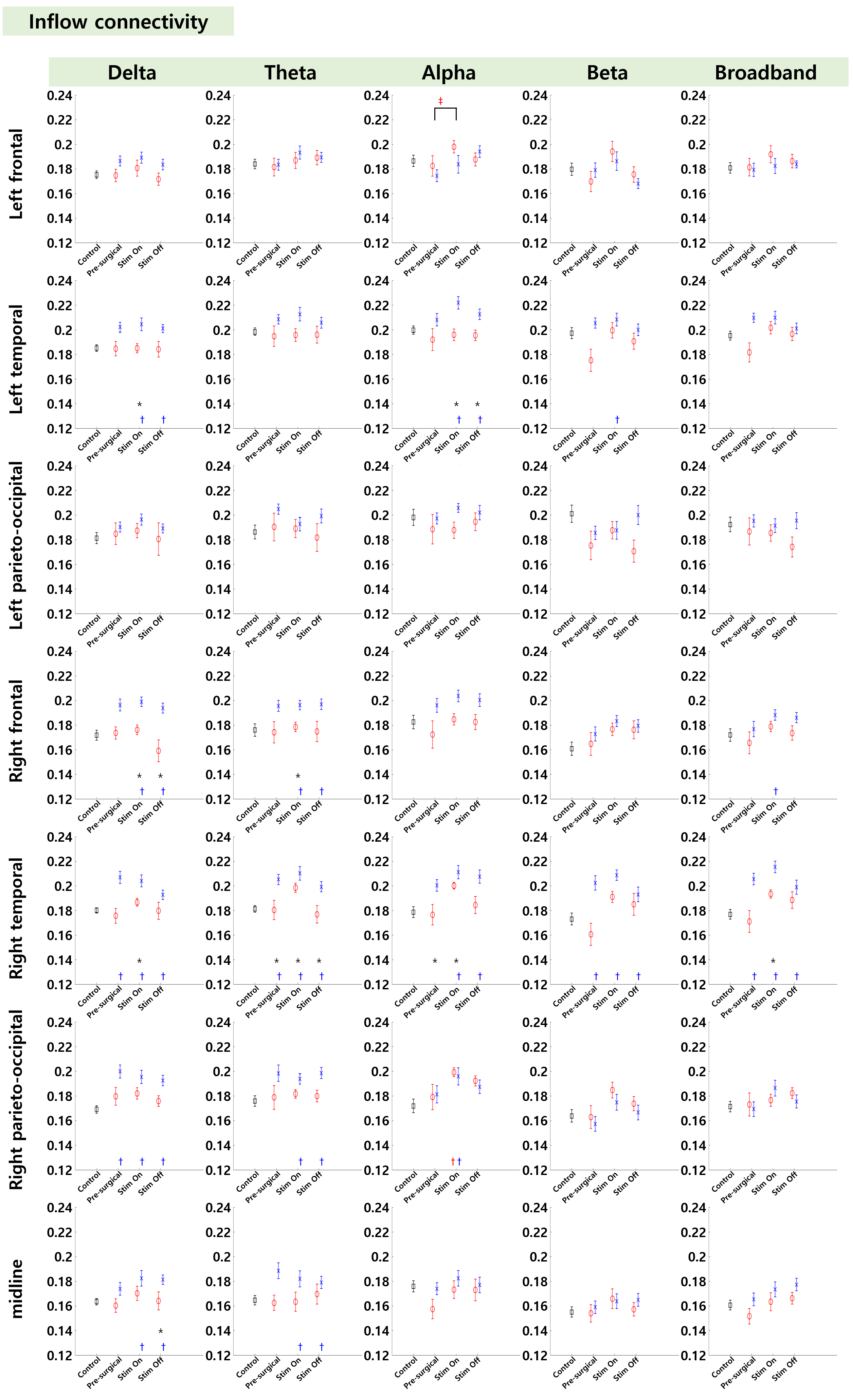

Supplement: Supplementary file 1 [file jcm-11-03695-s001.zip › Supplementary Figure S1 dotplot, inflow.tif]

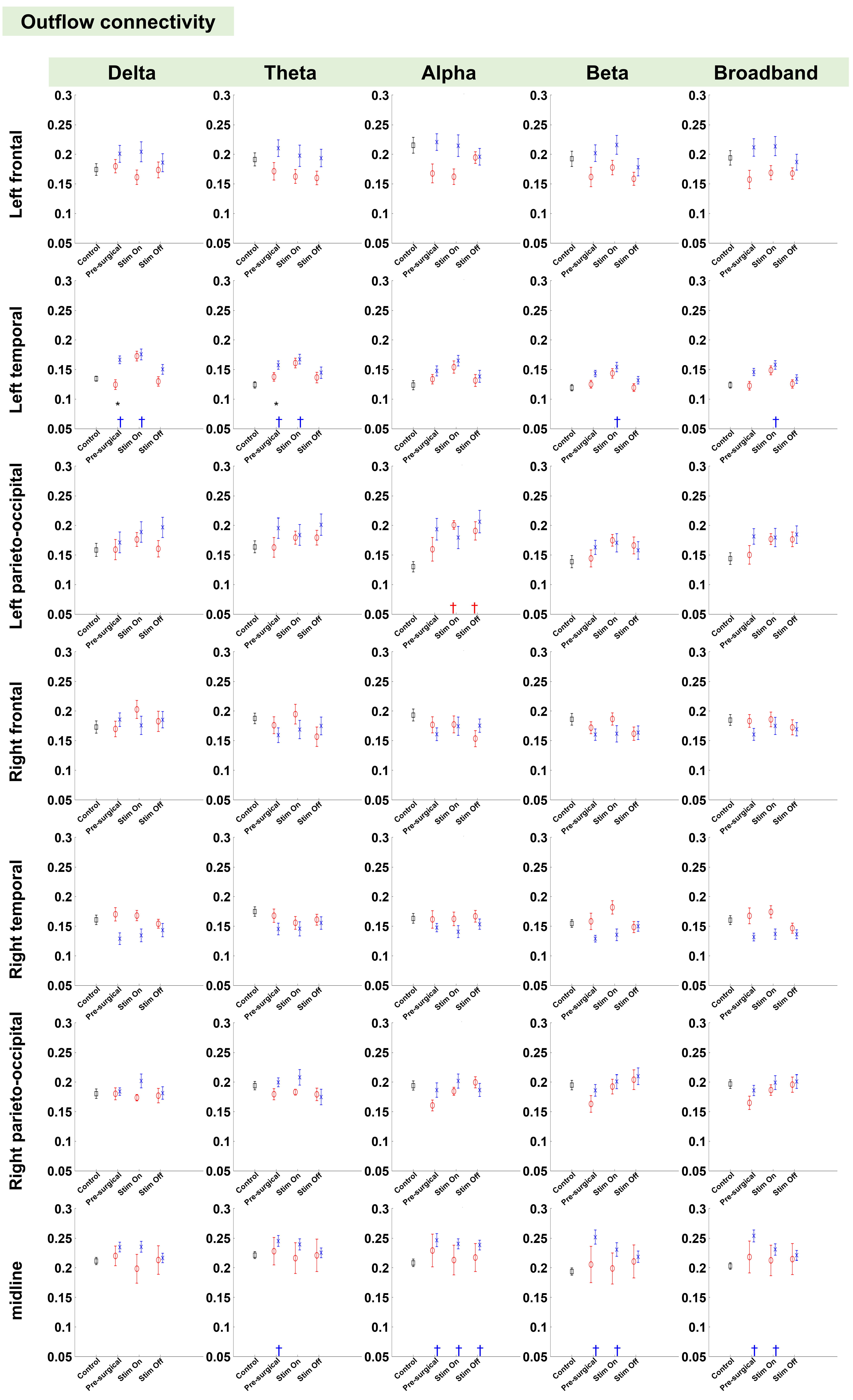

Supplement: Supplementary file 1 [file jcm-11-03695-s001.zip › Supplementary Figure S2 dotplot, outflow.tif]

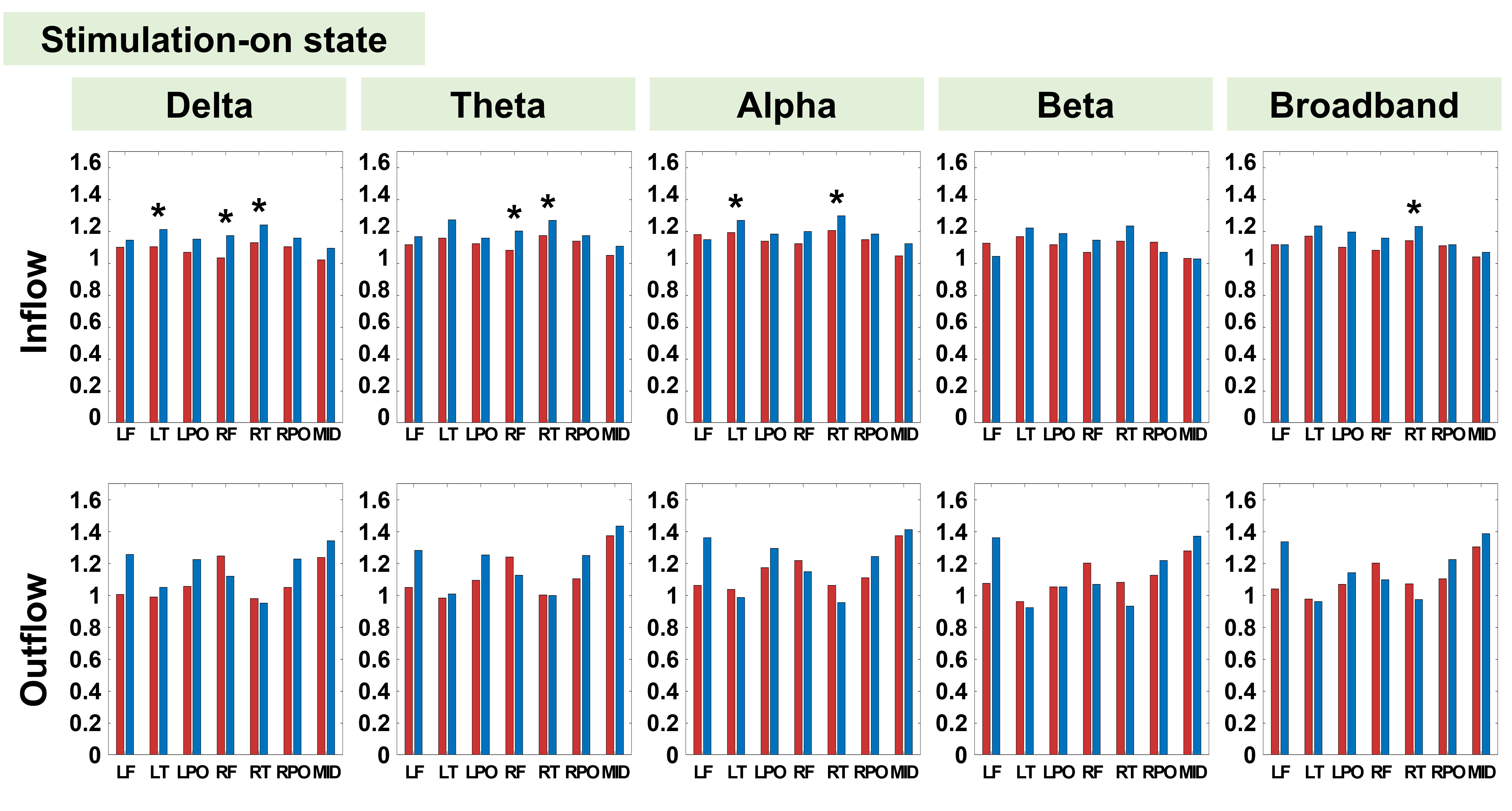

Supplement: Supplementary file 1 [file jcm-11-03695-s001.zip › Supplementary Figure S3 barplot, stimulation-on.tif]

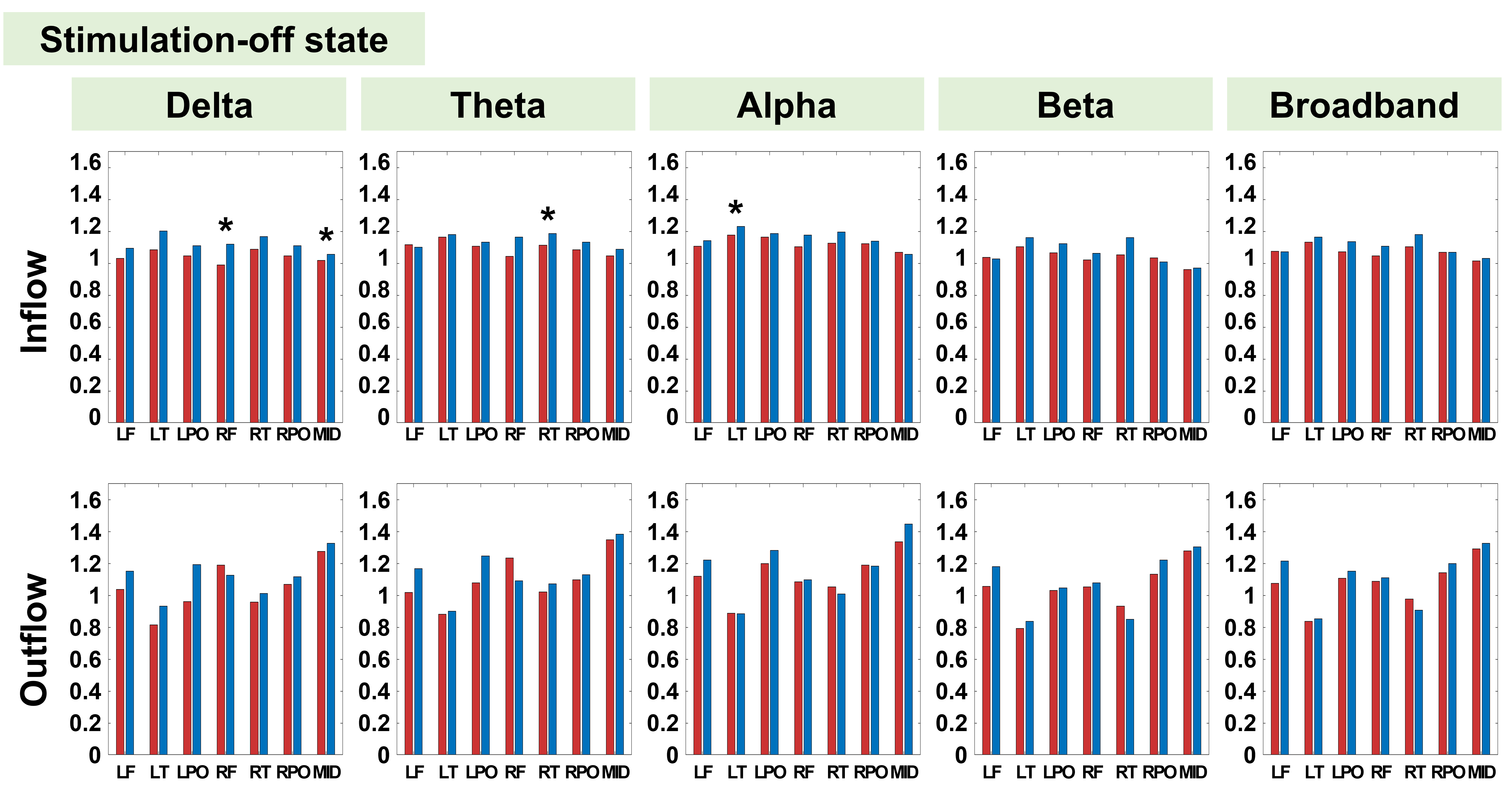

Supplement: Supplementary file 1 [file jcm-11-03695-s001.zip › Supplementary Figure S4 barplot, stimulation-off.tif]
